# Acute Anticholinesterase Pesticide Poisoning Caused a Long-Term Mortality Increase: A Nationwide Population-Based Cohort Study

**DOI:** 10.1097/MD.0000000000001222

**Published:** 2015-07-31

**Authors:** Hung-Sheng Huang, Chien-Chin Hsu, Shih-Feng Weng, Hung-Jung Lin, Jhi-Joung Wang, Shih-Bin Su, Chien-Cheng Huang, How-Ran Guo

**Affiliations:** From the Department of Emergency Medicine, Chi-Mei Medical Center, Tainan, Taiwan (H-SH, C-C Hsu, H-JL, C-C Huang); Department of Occupational Medicine, Chi-Mei Medical Center, Tainan, Taiwan (H-SH, S-BS, C-C Huang); Department of Biotechnology, Southern Taiwan University of Science and Technology, Tainan, Taiwan (C-C Hsu, H-JL); Department of Medical Research, Chi-Mei Medical Center, Tainan, Taiwan (S-FW, J-JW); Department of Healthcare Administration and Medical Informatics, Kaohsiung Medical University, Kaohsiung, Taiwan (S-FW); Department of Emergency Medicine, Taipei Medical University, Taipei, Taiwan (H-JL); Department of Leisure, Recreation, and Tourism Management, Southern Taiwan University of Science and Technology, Tainan, Taiwan (S-BS); Department of Medical Research, Chi Mei Medical Center, Liouying, Tainan, Taiwan (S-BS); Department of Environmental and Occupational Health, College of Medicine, National Cheng Kung University, Tainan, Taiwan (C-C Huang, H-RG); Department of Child Care and Education, Southern Taiwan University of Science and Technology, Tainan, Taiwan (C-C Huang); Department of Geriatrics and Gerontology, Chi-Mei Medical Center, Tainan, Taiwan (C-C Huang); Department of Occupational and Environmental Medicine, National Cheng Kung University Hospital, Tainan, Taiwan (H-RG)

## Abstract

Acute anticholinesterase pesticide (organophosphate and carbamate) poisoning (ACPP) often produces severe complications, and sometimes death. We investigated the long-term mortality of patients with ACPP because it is not sufficiently understood. In this retrospective nationwide population-based cohort study, 818 patients with ACPP and 16,360 healthy comparisons from 1999 to 2010 were selected from Taiwan's National Health Insurance Research Database. They were followed until 2011. Ninety-four (11.5%) ACPP patients and 793 (4.9%) comparisons died (*P* < 0.01) during follow-up. The incidence rate ratios (IRRs) of death were 2.5 times higher in ACPP patients than in comparisons (*P* < 0.01). The risk of death was particularly high in the first month after ACPP (IRR: 92.7; 95% confidence interval [CI]: 45.0–191.0) and still high for ∼6 months (IRR: 3.8; 95% CI: 1.9–7.4). After adjusting for age, gender, selected comorbidities, geographic area, and monthly income, the hazard ratio of death for ACPP patients was still 2.4 times higher than for comparisons. Older age (≥35 years), male gender, diabetes mellitus, coronary artery disease, hypertension, stroke, mental disorder, and lower monthly income also predicted death. ACPP significantly increased long-term mortality. In addition to early follow-up after acute treatment, comorbidity control and socioeconomic assistance are needed for patients with ACPP.

## INTRODUCTION

Organophosphates and carbamates are the most commonly used agricultural and household anticholinesterase pesticides.^1,2^ They inhibit cholinesterase activity, which overstimulates nicotinic and muscarinic acetylcholine receptors.^3–7^ The serious signs and symptoms of acute anticholinesterase pesticide poisoning (ACPP) are agitation, confusion, coma, respiratory failure, and sometimes death.^7^ Despite an awareness of the toxicity, the incidence of accidental, environmental, and occupational exposures and suicidal poisoning remain high, especially in developing countries, because anticholinesterase pesticides are effective and convenient.^1,2,7^

ACPP is a major global public health problem.^8–10^ In the Asia Pacific region, of the estimated 50,000 suicide deaths each year, about 60% are results of pesticide poisoning.^10^ Many studies have reported that ACPPs are responsible for about two-thirds of self-poisoning deaths.^11^ In spite of medical advances, the mortality of ACPP is still high, estimated at 12.7% to 30%.^5,11–13^

Zunec et al^14^ suggested that ACPP might increase lipid peroxidation and reactive oxygen species. Under high oxidative stress, cells typically undergo necrosis because of tissue damage, which can include subchronic and chronic toxicity and inflammation in various tissue types throughout the body.^15,16^ Some studies^1,7^ have been conducted but focus almost exclusively on predicting acute mortality and managing complications; however, the long-term prognosis of ACPP is still unclear. Therefore, we used Taiwan's National Health Insurance Research Database (NHIRD) to investigate, in a nationwide retrospective cohort study, the long-term mortality of patients with ACPP. We aimed to determine whether patients with ACPP have a higher mortality risk than do the general population because of the chronic toxicity of anticholinesterase pesticides and neurologic sequelae.

## METHODS

### Data Sources

The Taiwan National Health Insurance (NHI) Program is a universal healthcare system that covers nearly 100% of the country's population.^17^ The NHIRD contains all claims data from 1996 through 2011. This study used the Longitudinal Health Insurance Database 2000 (LHID2000), a subdataset of the NHIRD, which contains all claims data of 1 million (4.34% of the total population) beneficiaries who were randomly selected in 2000. The age, gender, and healthcare costs between the LHID2000 dataset and all NHI enrollees are not significantly different.

### Design

In this retrospective cohort study, we selected patients from the LHID2000 who had been newly diagnosed with ACPP (ICD-9 code 989.3) between January 1, 2002, and December 31, 2010 as the ACPP cohort. Members of the comparison cohort (without ACPP; 1:20 patient/comparison ratio) were randomly selected from the LHID2000 by matching age, gender, and index date (when ACPP was first diagnosed in the database) with the ACPP cohort.

**FIGURE 1:**
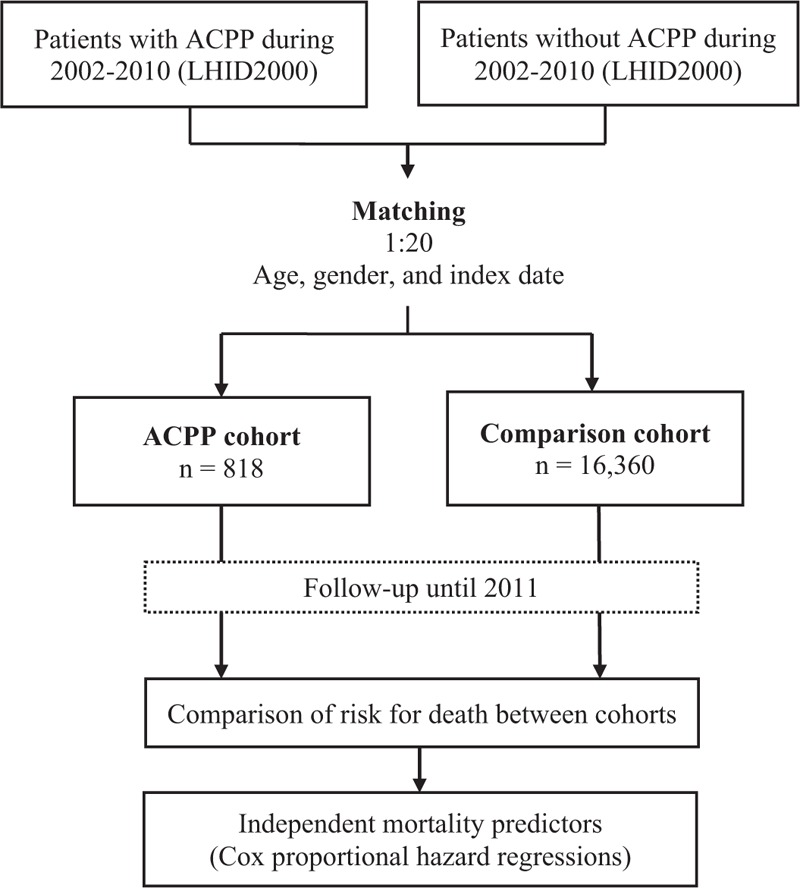
Flowchart of the study. ACPP = anticholinesterase pesticide poisoning, LHID2000 = Longitudinal Health Insurance Database 2000.

We linked to the diagnostic codes through the NHIRD and collected data including demographics, comorbidities, survival status, and date of death. Comorbidities affecting mortality that may have presented before the index date were defined as follows: diabetes mellitus (DM) (ICD-9 code 250), coronary artery disease (CAD) (ICD-9 codes 410–414), stroke (ICD-9 codes 430–438), hypertension (HTN) (ICD-9 codes 401–405), and mental disease (ICD-9 codes 290–319). We considered these to be comorbidities if they occurred either in the inpatient setting or in 3 or more ambulatory care claims coded before the index date. Patients were followed from the index date to the date of death or the end of the database period. All citizens in Taiwan are required to participate in the NHI, and their enrollment must be withdrawn within 30 days postmortem. Therefore, patients recorded as deceased or disenrolled within 30 days of their discharge were presumed dead, and the discharge date was designated as the date of death. Figure 1 shows a flowchart of this study.

### Ethics Statement

This study was conducted according to the Declaration of Helsinki. The Institutional Review Board at the Chi-Mei Medical Center approved this study and waived the need for informed consents from patients because the dataset consists of deidentified data. This waiver does not affect the rights and welfare of the patients.

### Statistical Analysis

We used Pearson χ^2^ tests for categorical variables and Student *t* test for continuous variables to compare the demographic characteristics and comorbidities^18^ and Poisson regression by calculating the incidence rate ratio (IRR) to compare the risk of death^18^ between ACPP cohort and Comparison cohort. The survival curves between 2 cohorts were compared by Kaplan–Meier analysis and the log-rank test.^18^ We used multivariate Cox proportional hazard regressions with adjustment for confounders^18^ to determine the effect of ACPP, age, gender, comorbidities, geographic region, and monthly income on the risk of death. We used SAS (Version 9.3.1 for Windows, SAS Institute, Inc., Cary, NC) for all the analyses in this study. Significance was set at *P* < 0.05 (2-tailed), except for the Poisson regression, in which a more conservative level of significance was set at 0.0033 (0.05/15) with Bonferroni correction because of the multiple comparisons performed.

## RESULTS

### Demographic Data

We recruited 818 patients with ACPP cohort and 16,360 age-, gender-, and index date-matched comparison cohort (Figure 1; Table 1) from the Taiwan LHID2000. The age (mean ± SD) in the both cohorts was 54 ± 16 years (Table 1). The majority of the enrollees were ≥50 years old (60%) and male (69%) in both cohorts, but neither age nor gender differences were significant. The ACPP cohort was significantly more likely to have comorbid stroke or a mental disorder than was the comparison cohort. In contrast, the ACPP cohort was significantly less likely to have a higher monthly income than was the comparison cohort.

**TABLE 1:**
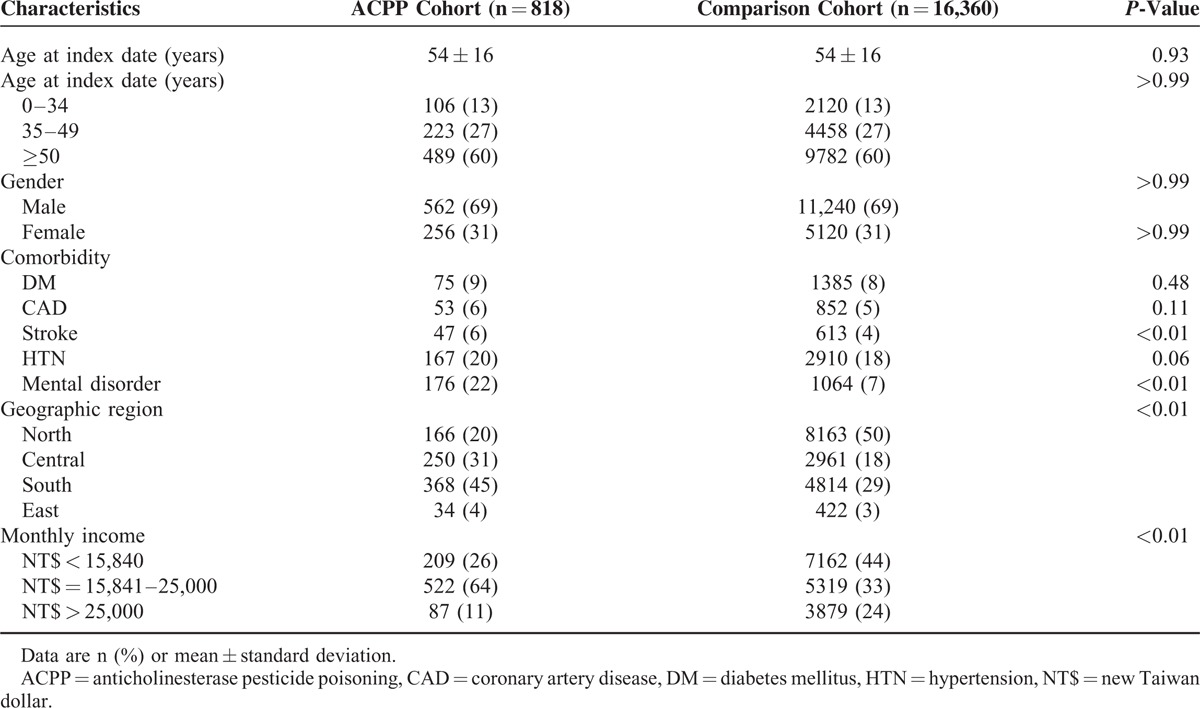
Demographic Characteristics and Comorbidities for ACPP Cohort and Comparison Cohort

### Risk of Death

**FIGURE 2:**
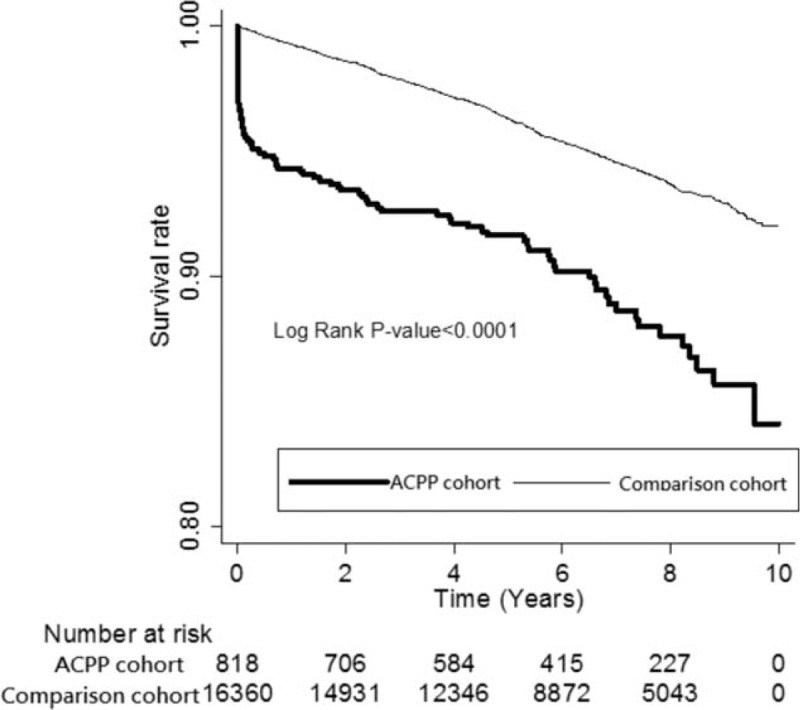
Survival rate for patients with anticholinesterase pesticide poisoning (ACPP) cohort and comparison cohort during the follow-up.

Overall mortality was 5.2% during the follow-up: 11.5% in the ACPP cohort and 4.9% in the comparison cohort (Table 2). The ACPP cohort had a significantly higher risk for death than did the comparison cohort (IRR: 2.5; 95% confidence interval [CI]: 2.0–3.1). The highest risk for death was in the first month after ACPP (IRR: 92.7; 95% CI: 45.0–191.0) and was still higher between 1 and 6 months (Table 2); however, there was no significant difference after 6 months. Kaplan–Meier survival analyses and log-rank tests also showed that the ACPP cohort had a significantly higher mortality risk than did comparison cohort during the follow-up period (Figure 2).

**TABLE 2:**
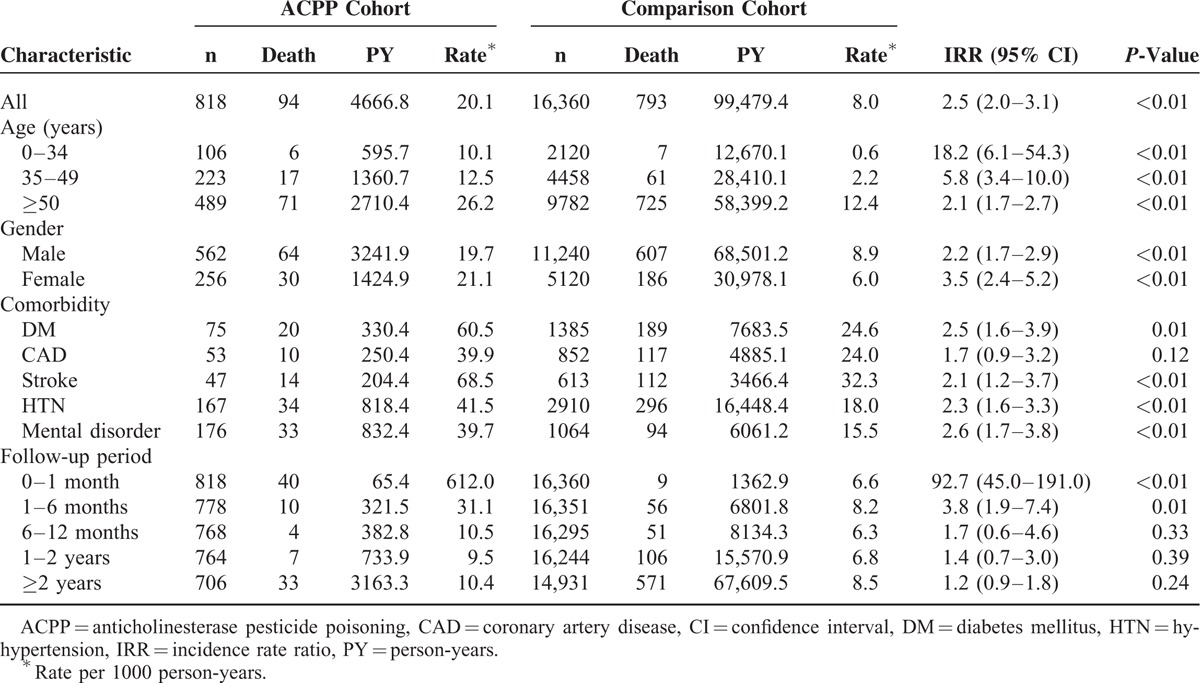
Risk of Death for ACPP Cohort and Comparison Cohort

The age subgroups of ACPP had higher IRRs than did their comparison counterparts (Table 2). ACPP cohort patients 0 to 34 years old had the highest risk for death (IRR: 18.2; 95% CI: 6.1–54.3) during the follow-up period, patients 35 to 49 had the second highest, and patients ≥50 had the third highest.

The mortality risk for both genders was significantly higher in the ACPP cohort than in the comparison cohort, especially for females (Table 2). The ACPP subgroups with comorbid DM, stroke, HTN, and mental disorder, but not CAD, had a higher risk of death than did their comparison subgroup counterparts (Table 2).

### Cox Proportional Hazard Regression

Cox proportional hazard regression was used to evaluate crude and adjusted hazard ratios (HRs) for death during the follow-up period. After patient age, gender, comorbidities, geographic region, and monthly income had been adjusted for, ACPP (adjusted HR: 2.4; 95% CI: 2.0–3.1) was still an independent predictor of mortality in all patients (Table 3), as were older age (≥35 years old), male gender, DM, CAD, stroke, HTN, mental disorder, and lower monthly income.

**TABLE 3:**
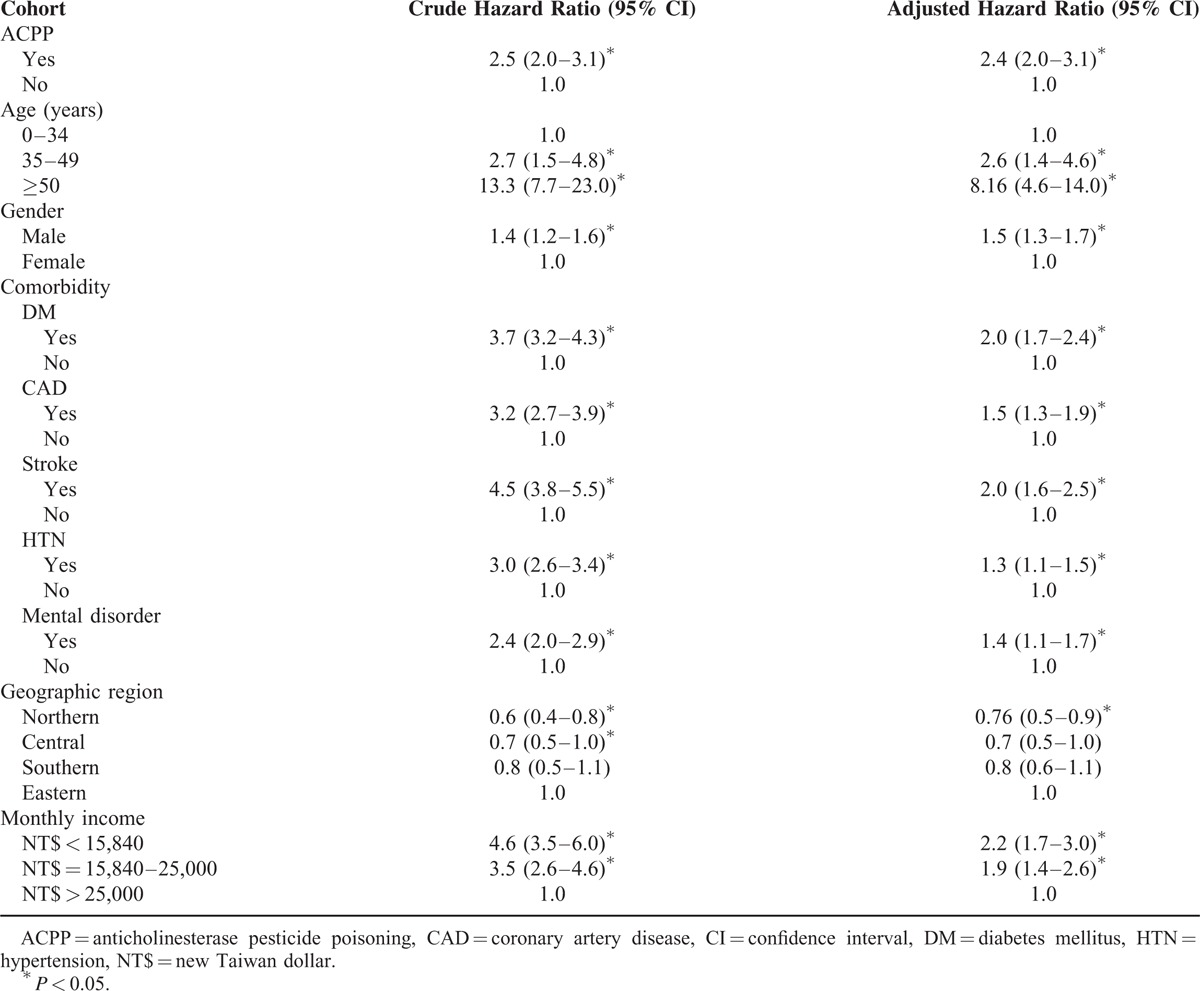
Crude and Adjusted Hazard Ratios of Cox Proportional Hazard Regressions and 95% Confidence Interval for Death During the Follow-Up

## DISCUSSION

This nationwide population-based cohort study showed that the long-term mortality risk increased in patients with ACPP, especially in the following subgroups: 0 to 34 years old, female, comorbid DM, comorbid stroke, comorbid HTN, and comorbid mental disorder. The IRR of death was significantly higher during the first month of follow-up after ACPP, and remained at 3.8 for the first 6 months. After potential confounders had been adjusted for, mortality in the ACPP cohort was still 2.4 times higher than in the comparison cohort. In addition, ≥35 years old, male, comorbid DM, comorbid CAD, comorbid stroke, comorbid HTN, comorbid mental disorder, and a monthly income <NT$ 15,840 were also independent mortality predictors. ACPP not only caused extremely high acute mortality within 1 month, but also increased long-term mortality for the first 6 months of follow-up. Early referral of patients with ACPP for a close follow-up, proper health education, better access to medical care, control of the comorbidities of DM, CAD, stroke, HTN, and mental disorder, and economic assistance may be urgently needed.

The increased long-term mortality risk after ACPP may be explained by the subacute and chronic tissue damage of oxidation and inflammation,^1,14–16^ and neurologic sequelae.^19^ Anticholinesterase pesticides are lipophilic and might accumulate in various tissues and organs after poisoning^1^ and subsequently be released into the bloodstream. A poisoning relapse may be prolonged and cause various clinical manifestations.^20^ Oxidative stress and inflammation may damage vascular walls, the liver, kidneys, pancreas, and so on.^1,15,16,21^ A recent study^1^ reported that ACPP increased the risk of deep vein thrombosis and pulmonary embolism. The authors suggested that inflammation might cause thrombotic tendencies and microvascular thrombosis by increasing procoagulant factors and inhibiting natural anticoagulant pathways.^1,21,22^ In response to chronic inflammation, the endothelium of the vascular wall may become dysfunctional with multiple outcomes, including the loss of anticoagulant, antiaggregant, and vasodilatory properties.^1,21,22^ These mechanisms eventually cause vascular thrombosis,^1^ other types of organ damage and complications, and even death. The cause of death for acute ACPP was mainly due to acute respiratory failure.^19^ Following an acute exposure, the patient may have neurologic sequelae^19^ such as hypoxic encephalopathy or persistent muscle weakness which may also increase the long-term mortality.

The majority of patients with ACPP were older (≥35 years) and male (Table 1); however, ACPP had a greater effect on mortality in younger (IRR: 0–34 years = 18.2, 35–49 = 5.8, ≥50 = 2.1) and female (IRR: males = 2.2, females = 3.5) patients (Table 2). The majority of older and male patients with ACPP corresponded to a study^12^ about organophosphate poisoning by Network of Taiwan's Poison Control Centers. The study recruited 4799 patients with organophosphate poisoning (mean age: 46 ± 18 years; 65.0% male; 65.0% suicide; mortality rate: 12.7%). Older people and men in Taiwan might have more opportunity than do others to use pesticide for suicide.^12^ Older age also predicted death for acute ACPP.^23^ The elderly are in poorer physiological condition and have altered toxicokinetics and toxicodynamics which predispose them to poor outcomes.^23^ However, younger and female patients may have fewer comorbidities; therefore, ACPP is one of the most likely factors that leads to death.

Comorbid DM, CAD, stroke, HTN, and mental disorder, and a monthly income <NT$ 15,840 predicted mortality. A recent hospital-based study^24^ reported that nearly half of the patients with ACPP had a history of a mental disorder (43.2%) and were undergoing long-term treatment with antipsychotic and antidepressant medications (25.4%). Another study^12^ in Taiwan reported that intentional exposure from suicide attempt, occupational exposure, and accidental exposure were 64.72%, 15.82%, and 13.27% in the patients with ACPP, respectively. In the United States, unintentional ACPP exposure (ie, accidental, occupational, and environmental) rarely resulted in mortality.^25^ On the other hand, several clinical studies^26^ suggested that chronic exposure to organophosphate pesticides might be associated with mental disorders. Whatever the reason that these comorbidities predict mortality after ACPP, the present study's results indicate that we have to manage not only the poisoning in patients with ACPP, but also the patients’ comorbidities and socioeconomic problems.

This study has some limitations. First, The NHIRD did not indicate the severity of the ACPP, did not specify whether the pesticide was an organophosphate or carbamate, did not specify the exposure route, and gave no detailed personal information like smoking status, body mass index, treatment (eg, atropine or pralidoxime therapy), or physical activity. All of these might be confounding factors. Second, misclassification of ACPP could not be completely avoided through using the ICD-9 codes in the claim records. It is possible that there were more cases of ACPP that were minor and treated, but not be correctly diagnosed. However, the consequence of such misclassification should be inclusion of ACPP patients in the comparison group and thus would tend to cause underestimation rather than overestimation of the relative risk. Therefore, our major conclusion that ACPP significantly increased long-term mortality should still be valid. Third, data on the cause of death, exposure types (eg, intentional, occupational, accidental, etc.), and association between death and exposure types were not available in this study, and therefore further studies to clarify related issues. For example, patients caused by intentional or occupational exposures may have higher mortality than those caused by accidental exposure. Fourth, this study could not evaluate whether there was a difference in long-term mortality between organophosphate and carbamate. Further studies on this issue are warranted. Fifth, our findings might not be generalizable to other nations.

## CONCLUSIONS

This first nationwide population-based cohort study on ACPP showed that it not only caused acute mortality within 1 month but also increased long-term mortality during the first 6 months after poisoning. ACPP had a greater effect on younger and female patients. Comorbid DM, CAD, stroke, HTN, and mental disorder, and a low monthly income were also independent mortality predictors. In addition to acute treatment, early follow-up and secondary mortality prevention are needed for patients with ACPP.
